# Prognostic factors for the improvement of pain and disability following multidisciplinary rehabilitation in patients with chronic neck pain

**DOI:** 10.1186/s12891-021-04194-9

**Published:** 2021-04-03

**Authors:** Martin Weigl, Josefine Letzel, Felix Angst

**Affiliations:** 1 Department of Orthopaedics, Physical Medicine and Rehabilitation, University Hospital, LMU Munich, Marchioninistr. 15, 81377 Munich, Germany; 2 Department of Internal Medicine, Klinikum Dritter Orden, Munich, Germany; 3 Research Department, Rehaklinik Bad Zurzach, Zurzach Care Group, Bad Zurzach, Switzerland

**Keywords:** Neck pain, Rehabilitation, Outcome assessment (health care), Pain clinics, Regression analysis, Prognosis

## Abstract

**Background:**

Recent clinical studies have demonstrated the effectiveness of specific, multidisciplinary, bio-psychosocial, rehabilitation programmes for chronic neck pain. However, prognostic factors for the improvement of pain and disability are mostly unknown. Therefore, the aim of this study was to explore prognostic factors associated with improvements in chronic neck pain following participation in a three-week, multidisciplinary, bio-psychosocial, rehabilitation programme.

**Methods:**

In this observational, prospective cohort study, a total of 112 patients were assessed at the beginning, end, and 6 months following the completion of a multidisciplinary, bio-psychosocial, rehabilitation programme. Inclusion for participation in the rehabilitation programme depended upon an interdisciplinary pain assessment. The primary outcome was neck pain and disability, which was measured using the Northern American Spine Society questionnaire for pain+disability and was quantified with effect sizes (ES). Multivariable linear regression analyses were used to explore potential prognostic factors associated with improvements in pain and disability scores at discharge and at the 6-month follow-up period.

**Results:**

The mean age of the patients was 59.7 years (standard deviation = 10.8), and 70.5% were female. Patients showed improvement in pain+disability at discharge (ES = 0.56; *p* < 0.001), which was sustained at the 6-month follow-up (ES = 0.56; *p* < 0.001). Prognostic factors associated with improvement in pain+disability scores at discharge included poor pain+disability baseline scores (partial, adjusted correlation r = 0.414, *p* < 0.001), older age (r = 0.223, *p* = 0.024), a good baseline cervical active range-of-motion (ROM) (r = 0.210, *p* < 0.033), and improvements in the Short-form 36 mental health scale (r = 0.197; *p* = 0.047) and cervical ROMs (r = 0.195, *p* = 0.048) from baseline values. Prognostic factors associated with improvements in pain+disability at the 6-month follow-up were similar and included poor pain+disability baseline scores (partial, adjusted correlation r = 0.364, *p* < 0.001), improvements in the Short-form 36 mental health scale (r = 0.232; *p* = 0.002), cervical ROMs (r = 0.247, *p* = 0.011), and better cervical ROM baseline scores. However, older age was not a factor (r = 0.134, *p* = 0.172).

**Conclusions:**

Future prognostic models for treatment outcomes in chronic neck pain patients should consider cervical ROM and mental health status. Knowledge of prognostic factors may help in the adoption of individualized treatment for patients who are less likely to respond to multidisciplinary rehabilitation.

**Supplementary Information:**

The online version contains supplementary material available at 10.1186/s12891-021-04194-9.

## Background

Neck pain is a musculoskeletal health condition with a high burden of disease. Previous epidemiological studies have reported a lifetime prevalence of the condition ranging from 14 to 71% [1]. The point prevalence of neck pain increases with age, until 45–49 years in females and 50–54 years in males, and then declines slowly, although it remains high [2]. A significant proportion of patients (37 to 47%) continue to suffer from neck pain one year following the onset of symptoms [3, 4].

Clinical guidelines and reviews recommend several treatment options for chronic neck pain (CNP); however, due to the small number of neck-pain-specific clinical trials, the evidence for beneficial interventions is weak to moderate [5–8]. Currently, the strongest evidence suggests that improvements can occur with local strengthening exercises and multi-modal exercises in the neck and shoulder region [9]. Other, less evidentiary, treatment options include mobilisation of the cervical or thoracic spine, aerobic exercises, patient education, and psychological interventions [5–9]. In a recent randomised control trial (RCT), as well as a clinical study that included the intra-individual control of effects, CNP-specific, multidisciplinary, bio-psychosocial rehabilitation (MBR) programmes were shown to improve pain and physical functioning for at least one year in patients who failed to respond to less complex interventions [10, 11]. However, our clinical experience suggests that individual responses to MBR vary considerably and may be dependent on specific prognostic factors.

Risk factors for developing neck pain include socio-demographic variables such as being female [3, 4], physical health factors including low endurance of the extensor muscles in the neck [12], and psychological factors such as depression [12].

Pre-treatment factors associated with poor outcomes include catastrophizing [13–16] and depressive symptoms [13, 16, 17], low-pain intensity in the neck and high-pain intensity in the upper extremities [18], the consumption of pain-related medication [14], and previous trauma [18]. Depressive symptoms as a prognostic factor has also been confirmed for several other musculoskeletal conditions [19, 20]. While occupational factors and the number of co-morbidities have been identified as prognostic factors for lower back pain [21, 22], we are unaware of any studies that have evaluated their association with outcomes after conservative, non-pharmacological treatment in CNP patients. In a study that included changes in co-variates as prognostic factors for CNP rehabilitation outcomes in whiplash injury patients, decreases in catastrophizing and depressive symptoms were associated with greater improvements in neck-pain-specific disability [17].

One recent systematic review included many studies that have been investigated prognostic factors for the course of disease in patients with neck pain [23]. However, they differed in patient populations (i.e. they involve acute, sub-acute, or chronic patients; those with previous whiplash injury; or patients with radicular pain), in the intervention (no intervention, a uni-modal or multi-modal intervention), or the applied outcome measure (global impression of change or patient questionnaires measuring pain and disability). Thus, it is unclear which prognostic factors may apply to the outcomes of pain and disability in CNP patients after MBR. None of the previous studies investigating prognostic factors has explored potential associations between changes in ranges of motion and patient reported outcomes for pain and disability.

More knowledge regarding prognostic factors associated with outcomes following an MBR programme in CNP patients is essential for adapting programme content to patients who show minimal improvement. Moreover, identifying associations between changes in physical and psychological health co-variates and outcomes would help to validate the existing content of MBR programmes.

Therefore, the objective of this study was to explore prognostic factors associated with improvements in pain and disability in CNP patients following their participation in an intensive, three-week, out-patient MBR programme. The primary aim was to investigate associations between changes in physical and psychological factors with pain and disability outcomes independently from baseline factors. The secondary aim was to advance the understanding of pre-treatment factors associated with outcomes.

Due to the bio-psychosocial treatment concept, we hypothesised that improvements in physical functioning and psychological health would be associated with less pain and disability.

## Methods

### Study design

A database from an observational prospective cohort study for the evaluation of outcomes from a CNP-specific MBR programme was analysed. Data were collected at an assessment before treatment (T0), at the beginning of treatment (T1), at the end of treatment (T2), at 6-months post-treatment (T3), and at 12-months post-treatment (T4). Due to the explorative nature of this study, we used all available data from time points T1, T2 and T3 and did not perform a sample size calculation. Moreover, we did not use the T4 data due to the high number of missing values for the relevant co-variates. Furthermore, the post-MBR programme effects at T4 were individually compared to patient health statuses from T0 and T1 in a previously published study [10].

The study was conducted at the day clinic in the Department of Physical Medicine and Rehabilitation at University Hospital, Ludwig Maximilian University, Munich, Germany. It was carried out in compliance with the protocols of the Helsinki Declaration of 2004. All participants provided signed, informed consent prior to study participation. The Ethics Committee at the medical faculty of the Ludwig Maximilian University Munich did not have any objections against the publication.

### Participants

Patients were referred to an interdisciplinary assessment at our clinic by a family physician or specialist. Depending on the primary diagnosis, patients were allocated to one of four condition-specific assessments for neck pain, lower back pain, osteoporosis, or osteoarthritis of the knee and hip. Assessments were conducted by a specialist in physical and rehabilitation medicine (PRM), a physiotherapist, and an occupational therapist. A psychologist from the treatment team also assessed patients who were suspected of suffering from a mental health disorder.

At the end of each assessment, the treatment team either recommended participation in a three-week MBR programme or another treatment option. The MBR programme was recommended according to predefined inclusion criteria, an appraisal of the results of standardised clinical tests and patient questionnaires, and the general impression of the day clinic treatment team. The predefined inclusion criteria for the neck-pain-specific MBR programme were: CNP lasting at least three months (with or without pain radiation in the upper limbs), previous out-patient physical therapy that did not result in improvement according to the patient, limitations in activities, and sufficient German language skills to follow the instructions of the MBR programme. Previous out-patient treatment was defined as conventional care for at least three months and typically included 3 × 6 sessions of physiotherapy for 20–30 min per session.

The MBR programme was not recommended if patients had severe somatic or mental illnesses that limited their ability to participate (e.G. *major* depression), acute neck trauma in the previous three months, former whiplash injury with proven structural damage, neurological deficits occurring within the previous three months, chronic neurological deficits that would have prevented participation in exercise interventions, dizziness or vertigo with unclear aetiology, diffuse idiopathic skeletal hyperostosis (DISH), shoulder abduction or flexion less than 90°, or patients undergoing a pension application.

After meeting with the treatment team, the physician explained the recommendation to each patient. In conversation, patients expressed their expectations and goals for treatment and their treatment preferences. Within the framework of participatory decision-making, the recommendation could change. More details regarding the assessment have been described elsewhere [10].

**Fig. 1:**
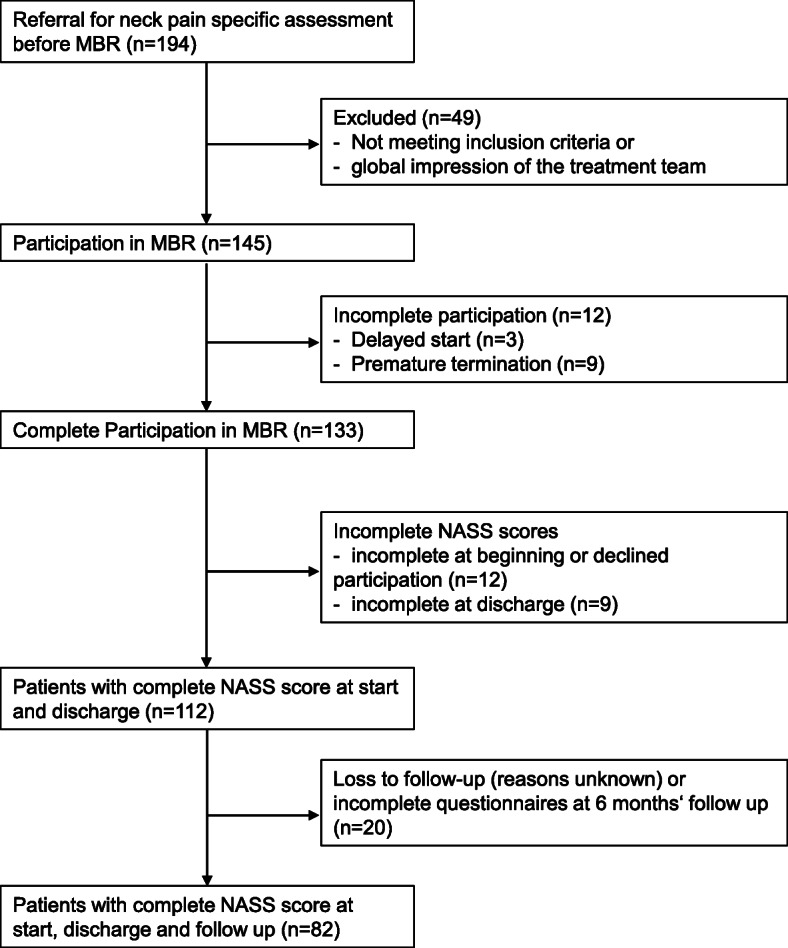
Patient flow diagram

All consecutive patients who participated in the entire MBR programme answered the North American Spine Society questionnaire (NASS) pain+disability scales [24, 25] at baseline and discharge. A study inclusion flow diagram is presented in Fig. 1.

### Data collection

At the beginning (T1) and the end (T2) of the MBR programme, patients completed a set of questionnaires and underwent standardised clinical tests. Each patient was assessed by the same trained physiotherapist or occupational therapist both times. Data was collected using the NASS questionnaire [24, 25], the mental health scale from the Short Form 36 (SF-36) questionnaire [26, 27], and a standardised co-morbidity questionnaire [28]. Socio-demographic information was also collected, and cervical spine range-of-motion (ROM) measurements were obtained using a cervical ROM instrument (CROM) [29]. At the 6-month follow-up (T3), the same questionnaires were sent to the patients by mail. Pre-addressed, stamped envelopes were provided to all participants.

### Study intervention

The clinic provides condition-specific, 3-week MBR programmes for patients with CNP, chronic lower back pain, osteoporosis and osteoarthritis of the knee and hip. Each programme alternates one after the other.

Patients completed a three-week, neck-pain-specific MBR programme that included a total of nine treatment days and 44 treatment hours. The programme fulfilled the German procedure classification (Operationen- und Prozedurenschlüssel (OPS)) 8–563.1 criteria of the German health care system, which requires at least 15 treatment units (a minimum of 30 min) of physical therapy or psychological therapy per week [30]. The OPS code, in combination with staying at least 6 h per day, is required for day clinic reimbursement by statutory health insurance in Germany.

The treatment team consisted of a specialist in PRM, physiotherapists, occupational therapists, psychologists, medical massage therapists, and a swimming trainer. Most treatments were provided to groups; although, all participants had two individual physiotherapy lessons that occurred at the beginning and end of the programme. During the initial, individual physiotherapy lesson, patients were trained in deep neck muscle strengthening exercises using biofeedback [31]. During the final individual lesson, patients were instructed in how to perform individual home exercises. Group treatments consisted of up to five participants in practical lessons and up to 10 participants in educational lessons and pool therapy. The MBR included land-based group exercises, gym training, pool exercises, occupational training, psychological lessons (including relaxation strategies), instructions for self-help techniques, patient education by a PRM specialist, and interactive group discussions at the end of each week with the entire treatment team. The physician provided daily ward rounds for the group, as well as individual appointments on demand. Details of the intervention have been described elsewhere [10].

### Measures

#### North American Spine Society questionnaire

The NASS is a condition-specific instrument with specific modules for lower back and neck pain [24, 25]. Its original version includes two scales that measure pain+disability and neurogenic symptoms that were derived from a principal factor analysis in the primary validation study. The cervical spine NASS pain+disability scale includes 11 items and the neurogenic symptom scale eight items. All items range from 1 (best health) to 6 (worst health). The scales were scored by calculating the arithmetic mean of the answers.

The German language version of the cervical spine NASS has demonstrated good criterion and discriminant validity, and sensitivity to change in validation studies [32, 33]. It has also shown good psychometric properties in a validation study conducted on patients who underwent intensive out-patient rehabilitation at health resorts [34]. Validated German versions of more commonly used cervical spine instruments, such as the Neck Disability Index (NDI), were not available at the time of the study; therefore, the cervical spine NASS was used.

Subsequent validation studies of the cervical spine NASS have shown a better fit in factor analyses and good responsiveness for separated scales for pain (two items) and disability (eight items) [32, 35]. In this study, we adhered to the original combined pain+disability scale as a primary outcome because it was defined a priori as a primary outcome in the evaluation of the neck-pain-specific MBR programme.

#### Short form 36 mental health

The SF-36 is the most widely used generic instrument for measuring health-related quality of life [26, 27]. Using 36 items, the following eight scales were determined: physical functioning, role-physical, bodily pain, general health, vitality, social functioning, role-emotional, and mental health. All of the scales ranged from 0 (worst health) to 100 (best health). The mental health scale, consisting of five items, was included in our analysis as it covers the construct of affective health, especially depression, with high validity [19]. The SF-36 mental health scale shows high rates of completeness, high reliability, and high sensitivity to change in the rehabilitation setting of CNP patients [34], and has shown associations with the course of pain for different chronic conditions, including neck pain [19].

#### Cervical range-of-motion instrument

A CROM instrument (Performance Attainment Assoc., St. Paul, MN, USA) was used to measure the cervical spine ROM in degrees. The instrument consists of a mounting device for the head, two gravity-dependent goniometers, and a compass that measures rotation in 2° increments. A validation study showed good intra- and inter-tester reliability [29], and reliability and validity has been confirmed in different populations [36]. The minimal detectable change in each direction was between 3.6° and 9.3° [37, 38]. The total active cervical ROM was the sum of 6 directions and showed higher intra- and inter-observer reliability (ICC = 0.99 and 0.95, respectively) compared to each separate cervical measure [39]. The standard errors of the mean for the intra- and inter-observer studies were 6.6 and 17.7°, respectively [39]. We used the total active cervical ROM rather than the separate measures due to limitations in the number of co-variates used in the analysis.

#### Socio-demographic data and co-morbidities

Information concerning co-morbidities was collected using the standardized Self-Administered Comorbidity Questionnaire (SCQ) [28], and socio-demographic data was gathered through specific questions.

### Analyses

#### Descriptive statistics and treatment effects

Descriptive statistics were calculated for the baseline characteristics. Effect sizes (ESs) for the primary outcome (NASS pain+disability) and the secondary outcomes (NASS pain, NASS disability, SF-36 mental health, and the total active cervical ROM) were determined by dividing the mean change between baseline and discharge (T1 and T2), and between baseline and the 6-month follow-up (T1 and T3), by the standard deviation of the baseline score [40]. An ES above 0.30 is generally considered to be clinically meaningful unless instrument-specific studies have provided more reliable results for the minimal clinically important effects [41]. For the NASS pain+disability scale, no specific minimal clinically important differences have been previously quantified. Significance of changes were tested using t-tests for dependent samples of normally distributed data or with Wilcoxon signed-rank tests for non-normally distributed data.

#### Multivariable regression

In the primary exploratory linear regression models, the dependent variables were ∆ discharge – baseline, and ∆ 6-month follow-up – baseline of the NASS pain+disability scale. For additional linear regression modelling, the dependent variables were the ∆ discharge – baseline of the separated NASS pain and disability scales.

Independent variables were selected from candidate variables in the database and based on previous research concerning risk factors and prognostic factors in neck pain patients [12–20, 22, 42, 43], as well as on clinical experience. We further aimed to cover both physical and psychological health and were specifically interested in the change in the ROM co-variable, which was a treatment aim of the MBR programme [10] and is associated with pain and disability in CNP patients [44].

The analyses were adjusted for the baseline variables of the change scores and important socio-demographic characteristics. The total number of co-variates was limited to 10, as 10 cases per co-variate were needed for the finite models and sufficiently valid estimates of the regression coefficient [45].

The independent variables were sex, age, living with a partner, education level, number of co-morbidities, SF-36 mental health baseline value and mean change, the total active cervical ROM, and the change in the total active cervical ROM. All models were adjusted for the baseline score of the corresponding NASS scale, of which the change in score was the dependent variable. To adjust for any confounding, all listed co-variates were kept in the models, irrespective of whether their correlation was statistically significant. Multivariable partial correlations were determined and adjusted for all other potentially confounding co-variates [46]. The overall explained variances (%) were calculated to quantify the fit of the regression models.

For missing values of single co-variates, the mean imputation method was used (i.e. missing values were replaced with the mean of the valid values within each independent variable). This method was provided by the linear regression module of the statistical software program SPSS 25.0. Imputation by linear regression would have been inappropriate as it was the same strategy as the evaluation of prognostic factors and, therefore, would have increased the number of valid cases, but not the outcome of the prognostic parameter estimates. We assumed missing values were due to random processes, as the main reasons for missing data were incomplete distributions of the questionnaire regarding socio-demographic characteristics (12 patients = 10.7%) and incomplete clinical tests at T1 or T2 (10 patients = 8.9%) and not due to refusals by the patients to fill-in questionnaires or undergo clinical tests. This assumption was further supported by 97% completeness of the SF-36 mental health scale at baseline and 98% at follow-up, despite patients typically viewing questions about mental health as more sensitive compared to those concerning physical health.

All statistical analyses were calculated using SPSS 25.0 for Windows (IBM Corp., Armonk, NY, USA). ESs were calculated using Microsoft Excel 2010.

## Results

### Participant characteristics and baseline scores

Table 1 summarises the socio-demographic characteristics of 112 patients included in the study from March 2006 to June 2012. The mean patient age was 59.7 years (SD: 10.8; min = 29.7; max = 81.3), and a majority were female (71%). Approximately 40% of the patients had three or more co-morbidities and only 12% had no co-morbidities. Half of the patients (50%) had at least a high school diploma.

**Table 1:** Socio-demographic characteristics of the study population (*n* = 112)

Characteristic	Value
Female, *n* (valid %)	79 (70.5)
Age (years), mean (SD)	59.7 (10.8)
Living with a partner, *n* (valid %),	68 (69.7)
Missing, *n*	12
Education, *n* (valid %)	
Basic school	10 (10.4)
Vocational training	38 (39.6)
High school	18 (18.8)
Technical college	12 (12.5)
University	18 (18.8)
Missing, *n*	16
Co-morbidities, *n* (valid %)	
None	12 (12.1)
1	20 (20.2)
2	27 (27.3)
3	22 (22.2)
≥ 4	18 (18.1)
Missing, *n*	13

SD: standard deviation

### Treatment outcomes

At discharge (T2), patients showed better scores in comparison to baseline in all outcomes: NASS pain+disability (ES = 0.56, *p* < 0.001), NASS pain (ES = 0.67, *p* < 0.001), NASS disability (ES = 0.41, *p* < 0.001), SF-36 mental health (ES = 0.45, *p* < 0.001), and total active cervical ROM (ES = 0.39, *p* < 0.001) (Table 2).

**Table 2:** Outcome scores at discharge (T2) (*n* = 112)

	Entry		Discharge		Entry ➔ Discharge ES	*p*-value
	Mean	SD	Mean	SD		
NASS						
pain+disability	2.80	0.71	2.40	0.68	0.56	< 0.001
pain	4.30	1.12	3.56	1.15	0.67	< 0.001
disability	2.41	0.74	2.11	0.70	0.41	< 0.001
SF-36 mental health	64.7	19.5	73.5	15.5	0.45	< 0.001
Total active ROM (°) *	236.1	44.5	253.6	40.6	0.39	< 0.001

*Total active ROM: sum of the range-of-motion for cervical lateral flexion (both sides), cervical rotation (both sides), neck flexion, and neck extension. NASS: North American Spine Society questionnaire (1 = best health; 6 = worst health); SF-36: Short Form 36 questionnaire (0 = worst health; 100 = best health); ES: effect size

At the 6-month follow-up (T3), improvements in the NASS scores were sustained with ES levels similar to those at discharge (T2) (Table 3). The ES for the SF-36 mental scale decreased from 0.45 to 0.18. The total active ROM was not tested at T3.

**Table 3:** Outcome scores at the 6-month follow-up (*n* = 82)

	Entry		6 months		Entry ➔ 6 months ES	*p*-value
	Mean	SD	Mean	SD		
NASS						
pain+disability	2.76	0.73	2.35	0.76	0.56	< 0.001
pain	4.28	1.12	3.32	1.30	0.86	< 0.001
disability	2.37	0.75	2.11	0.79	0.35	< 0.001
SF-36 mental health	66.4	18.9	69.8	16.6	0.18	0.033

NASS: North American Spine Society questionnaire (1 = best health; 6 = worst health); SF-36: Short Form 36 questionnaire (0 = worst health; 100 = best health); ES: effect size

### Prognostic factors associated with short-term changes in pain and disability

Univariable associations between the co-variates and changes in the NASS pain+disability score are presented in Table 4. Changes in the SF-36 mental health scale were associated with improvements in pain+disability, with an unadjusted correlation of r = 0.219. Among the baseline variables, the NASS pain+disability score showed a moderate unadjusted correlation, with r = 0.376. All other co-variates showed correlations smaller than r = 0.200.

**Table 4:** Multivariable regression of changes in NASS pain+disability scores between baseline and programme discharge (*n* = 112)

Covariate	Change R^2^	Change F-value	Regression coefficient	95% CI	*p*-value	Bivariate correlation	Partial correlation
Constant			−1.890	(−2.974 to 0.805)	0.001		
NASS pain+disability baseline	0.151	2.209	0.281	(0.159 to 0.403)	< 0.001	0.376	0.414
Age	0.038	0.312	0.009	(0.001 to 0.017)	0.024	0.077	0.223
Active ROM,* baseline	0.034	0.228	0.002	(0.000 to 0.005)	0.033	0.004	0.210
SF-36 mental health, change	0.029	0.140	0.007	(0.000 to 0.013)	0.047	0.219	0.197
Active ROM,* change	0.029	0.132	0.003	(0.000 to 0.006)	0.048	0.052	0.195
SF-36 mental health, baseline	0.024	0.030	0.005	(0.000 to 0.010)	0.072	−0.085	0.178
Education	0.018	−0.093	−0.050	(−0.113 to 0.013)	0.121	−0.158	− 0.154
Sex (0 = female; 1 = male)	0.006	−0.336	− 0.079	(− 0.250 to 0.093)	0.365	− 0.017	− 0.090
Marital Status (1 = alone; 2 = with partner)	0.003	− 0.406	0.053	(−0.122 to 0.228)	0.546	0.087	0.060
Co-morbidities	0.002	−0.410	0.018	(−0,043 to 0.079)	0.560	0.172	0.058
Model total	0.273	3.784			0.000		

* Active ROM: sum of the ranges-of-motion of cervical lateral flexion (both sides), cervical rotation (both sides), neck flexion, and neck extension. Positive regression coefficients for change scores represent positive associations. NASS: North American Spine Society questionnaire; SF-36: Short Form 36 questionnaire

In the multivariable regression model, the change in the NASS pain+disability scale between baseline (T1) and discharge (T2) was modelled with 10 co-variates that explained 27.3% of the variance (Table 4). Positive and statistically significant correlations were found for the changes in the SF-36 mental health scale (partial, adjusted correlation: 0.197; *p* = 0.047), the total active ROM (0.195, *p* = 0.048), age (0.223, *p* = 0.024), and for poor NASS pain+disability baseline scores (0.414, *p* < 0.001).

Additional regressions were used to model changes in NASS disability and pain as dependent variables and are presented in additional files 1 and 2. A poor NASS disability score at baseline (partial, adjusted correlation: 0.42, *p* < 0.001), older age (0.25, *p* = 0.010), and a high total active ROM at baseline (0.25, *p* = 0.012) were all correlated with improved NASS disability at programme discharge (T2). However, only a high baseline NASS pain score was significantly correlated with pain relief on the NASS pain scale at T2 (partial, adjusted correlation: 0.36, *p* < 0.001).

### Prognostic factors associated with changes in pain and disability at the 6-month follow-up

Univariable associations between the co-variates and changes in the NASS pain+disability score between baseline (T1) and the 6-month follow-up (T3) are presented in Table 5. Improvement in SF-36 mental health was associated with improvements in pain+disability scores (unadjusted correlation r = 0.232). The NASS pain+disability baseline score showed the strongest correlation with improvement at T3 (r = 0.248). All other co-variates showed correlations smaller than 0.200.

**Table 5:** Multivariable regression of change in NASS pain+disability scores between baseline and the 6-month follow-up (*n* = 82)

Covariate	Change R^2^	Change F-value	Regression coefficient	95% CI	*p*-value	Bivariate correlation	Partial correlation
Constant			−1.658	(−2.821 to 0.495)	0.006		
NASS pain+disability baseline	0.120	1.964	0.261	(0.132 to 0.390)	< 0.001	0.248	0.364
SF-36 mental health, change	0.076	1.133	0.011	(0.004 to 0.018)	0.002	0.232	0.297
Active ROM,* change	0.051	0.613	0.004	(0.001 to 0.008)	0.011	0.173	0.247
Active ROM,* baseline	0.034	0.249	0.002	(0.000 to 0.005)	0.037	−0.020	0.204
SF-36 mental health, baseline	0.026	0.075	0.005	(0.000 to 0.010)	0.066	−0.077	0.180
Age	0.014	−0.195	0.006	(−0.002 to 0.014)	0.172	−0.012	0.134
Sex (0 = female; 1 = male)	0.007	−0.363	0.086	(−0.087 to 0.259)	0.331	0.072	0.096
Education	0.002	−0.494	−0.016	(− 0.082 to 0.050)	0.632	− 0.043	−0.047
Model total	0.213	3.483			0.001		

* Active ROM: sum of the range-of-motion of cervical lateral flexion (both sides), cervical rotation (both sides), neck flexion, and neck extension. The change in active ROM refers to the change between baseline and discharge, as no mobility was measured after 6 months. Positive regression coefficients for change scores represent positive associations. NASS: North American Spine Society questionnaire; SF-36: Short Form 36 questionnaire

The regression model explained 21.3% of the variance (Table 5). Due to a smaller sample size (82 patients), only eight of ten co-variates were included in the model. The co-morbidity and marital status co-variates were deleted from the model in a backwards elimination procedure where baseline values of change variables were forced to stay in the model.

Improvements in the active cervical ROMs between T1 and T2 (partial, adjusted correlation 0.247, *p* = 0.204) and in the SF-36 mental health score between T1 and the T3 (0.297, *p* = 0.002) were significantly correlated to improvements in NASS pain+disability between T1 to T3. Furthermore, poor baseline NASS pain+disability scores (0.364, *p* < 0.001) and total active ROM (0.204, *p* = 0.037) were associated with a better outcome at the 6-month follow-up.

No significant correlations were found for sex, education, marital status, co-morbidities or the baseline SF-36 mental health score in any of the regression models.

## Discussion

This prospective cohort study investigated prognostic factors for both immediate and long-term changes in pain and disability following a CNP-specific MBR programme. Improvements in mental health from baseline at discharge and at the 6-month follow-up, and improvement in active cervical ROMs between baseline and discharge, were independently associated with a better outcome. Among the baseline variables, poor scores on the NASS pain+disability scale and poor active cervical ROMs correlated with improvements in pain+disability. In contrast to the discharge results, age was not a prognostic factor 6-months post-baseline. Furthermore, mental health at baseline, co-morbidities, level of education, sex and marital status showed no significant association with the treatment outcome.

The positive, longitudinal association between improvement in mental health and improvement in NASS pain+disability scores is in line with previous research. A recent cohort study found moderate to strong positive associations between improvements in mental health and pain relief for patients with neck pain after whiplash injury, knee osteoarthritis, lower back pain, fibromyalgia and lipedema. The authors of that study also reported a weak correlation between changes in mental health and pain following shoulder arthroplasty [19]. Moreover, whiplash patients in that study showed stronger associations compared to the CNP patients in our study (6 months’ follow-up adjusted correlations: r = 0.515 versus r = 0.232). This difference could be due to the worse mental health baseline status of the whiplash patients compared to our CNP patients, as correlations between improvements in mental health and pain tend to be stronger for patients with a worse baseline mental health status [19].

To our knowledge, this is the first study to evaluate the association between improvements in cervical spine ROMs and self-reported pain and disability during a comprehensive rehabilitation intervention. A reliable measure of cervical spine ROMs allows clinicians and researchers to evaluate the outcome and importance of neck-specific exercises in an MBR programme. However, in this study, the small improvements in the ROMs, and their weak correlations with a better outcome, do not allow any firm conclusions to be drawn.

A high baseline NASS score, reflecting more pain or disability, was a strong prognostic factor for better outcomes. This is a well-known phenomenon in studies of prognostic factors following multidisciplinary treatment of patients with chronic musculoskeletal pain [17, 42]. A regression toward the mean may have contribute to this association, as patients with very poor baseline scores have more room for improvement in comparison to those with better baseline scores [47].

Another prognostic factor was poor cervical ROM at baseline. This finding is similar to the results of a previous study investigating neck pain patients who underwent a multi-modal treatment that combined manipulation, exercise and patient education [48]. In that study, a cervical extension < 30 degrees was associated with a better outcome at the end of treatment. These results suggest that patients with a poor ROM at baseline may benefit more than others from multi-modal interventions that include treatments aimed at improving the cervical ROM.

The lack of association between age and long-term outcomes for pain and disability is consistent with two previous studies that have investigated prognostic factors in neck pain patients following multi-modal [14] or physical therapy [16]. Similar to our study, patients from all adult age groups were included in those studies. However, two previous reports on CNP patients in the working population (age < 65 years) found better long-term improvements for pain [17, 49] and disability [17] in younger patients. Thus, it is possible that age is a prognostic factor in working adults, but not in other populations. However, the current evidence does not allow drawing firm conclusions.

Mental health at baseline was associated with a change in pain and disability, and consistently showed relatively weak correlations (r ≤ 0.180) at both follow-ups (i.e. there was a trend, but it was not statistically significant). In one study that investigated whiplash injury patients receiving a multi-modal intervention, the association at the 6-month follow-up period was stronger (r = 0.368; *p* < 0.001) [17]. However, the patients in that study had worse mental health statuses compared to our patient population. In another study, researchers investigated neck pain patients receiving physical therapy and found a non-significant trend for a poor outcome in patients with poor mental health after 6 months (OR = 0.79, CI 0.58–1.08; *p* < 0.1) [16].

Baseline co-morbidities did not correlate with treatment outcomes. To our knowledge, this potential prognostic factor had not been previously evaluated in CNP patients. However, for musculoskeletal health conditions including lower back pain and osteoarthritis of the knee and hip, a low number of co-morbidities has been associated with better outcomes [20, 22]. Discrepancies with these studies may be explained by differences in patient health conditions, patient selection, treatments, outcome measures or statistical analyses. Furthermore, our results are similar to a previous study investigating CNP patients following multidisciplinary treatment, whereby the level of education and sex was not associated with the outcome [49]. Taken together, the lack of association for age, education, sex and marital status with the outcome pain+disabilty 6 months after treatment suggests that demographic and social factors are less relevant prognostic variables compared to psychological or physical health ones for treatment outcomes in CNP patients.

One strength of this study was the generalizability of the results to similar MBR programmes. In contrast to most RCTs, this research applied no artificial restrictions to participation or made adaptations to the intervention, which is routinely reimbursed by German statutory health insurance. Another strength concerns the application of a primary outcome measure that was both reliable and valid and included the International Classification of Functioning, Disability and Health (ICF) components of body function, activity and participation [32–34, 50, 51]. Moreover, the independent variables also covered the ICF components of mental function (SF-36 mental health), physical function (ROM and baseline NASS score), activity and participation (baseline NASS score), and personal factors (age, sex, education).

One limitation of this study was the relatively low levels of explained variance. This indicates that some important prognostic factors for pain and functioning were not assessed. In particular, potential prognostic occupational context factors [21, 43] or catastrophizing [13–16] was not available in the database. Adding additional context factors may increase the R-squared value and result in a higher number of prognostic factors. Nonetheless, these omissions do not affect the conclusions drawn from the significant correlations.

Another limitation concerns the loss to follow-up. There were 145 patients who began the MBR programme, yet 23 and 43% were not included at discharge and the 6-month follow-up, respectively. Although loss to follow-up is less problematic in a prognostic factors study compared to an efficacy study, it may have influenced the associations found in this study.

A further limitation was the absence of a control group. The Hawthorne effect refers to individuals modifying their behaviour in response to an awareness of being observed [52]. In this study, the intensive treatment and chronic complaints could have contributed to a substantial Hawthorne effect. However, the patients participated in an established treatment program and may have felt less observed than in an experimental study. If differences in the Hawthorne effect between individual patients were associated with the outcome, this could be a main cause for the low level of explained variance. However, it should not affect the reliability of the identified prognostic factors.

## Conclusions

Future prognostic models for treatment outcome in CNP patients should consider cervical ROM and mental health factors. Given large samples sizes, subsequent studies should include additional psychological variables such as catastrophizing, occupational factors, and cognitive-behavioural co-variables including coping styles and self-efficacy, in multivariable analyses. Knowledge of prognostic factors may help in the adoption of individualized treatment for patients who are less likely to respond to MBR programmes.

## Supplementary Information

**Additional file 1: Table.** Multivariable regression of the changes in NASS disability between baseline and programme discharge (*n* = 112)

**Additional file 2: Table.** Multivariable regression of the changes in NASS pain between baseline and programme discharge (*n* = 112)

## Data Availability

The data set is not available because patients did not consent to the use of their data in a public repository.

## Abbreviations

CNP: Chronic neck pain.
MBR: Bio-psychosocial rehabilitation.
ROM: Range-of-motion.
CROM: Cervical range-of-motion.
NASS: North American Spine Society questionnaire.
SF-36: Short Form 36.
SCQ: Self-Administered Co-morbidity Questionnaire.
ICF: International Classification of Functioning, Disability and Health.
