# Supplementary material for: Prognostic factors for the improvement of pain and disability following multidisciplinary rehabilitation in patients with chronic neck pain
Source: BMC Musculoskelet Disord. 2021 Apr 3;22:330. doi: 10.1186/s12891-021-04194-9 (PMC8019506; doi:10.1186/s12891-021-04194-9)
Supplement: Supplementary file 1 — Additional file 1: Table. Multivariable regression of the changes in NASS disability between baseline and programme discharge (n = 112) [file 12891_2021_4194_MOESM1_ESM.docx]

**Additional file 1:** Multivariable regression of change in NASS disability between baseline and programme discharge (*n*=112)

| Covariate | Change R^2^ | Change F-value | Regression coefficient | *p*-value | Bivariate correlation | Partial correlation |
| --- | --- | --- | --- | --- | --- | --- |
| Constant |  |  | -1.891 | <0.001 |  |  |
| NASS disability baseline | 0.161 | 2.248 | 0.264 | <0.001 | 0.37 | 0.42 |
| Age | 0.051 | 0.557 | 0.010 | 0.010 | 0.14 | 0.25 |
| Active ROM* baseline | 0.049 | 0.516 | 0.003 | 0.012 | 0.04 | 0.25 |
| Active ROM* change | 0.029 | 0.150 | 0.003 | 0.052 | 0.01 | 0.19 |
| SF-36 mental health, change | 0.015 | -0.116 | 0.005 | 0.162 | 0.19 | 0.14 |
| SF-36 mental health, baseline | 0.011 | -0.192 | 0.003 | 0.231 | -0.10 | 0.14 |
| Education | 0.004 | -0.317 | -0.024 | 0.443 | -0.05 | -0.08 |
| Marital Status (1=alone; 2=with partner) | 0.002 | -0.358 | 0.048 | 0.573 | 0.08 | 0.06 |
| Gender (0=female; 1=male) | 0.000 | -0.398 | -0.018 | 0.832 | 0.05 | -0.02 |
| Comorbidities | 0.000 | -0.399 | 0.006 | 0.842 | 0.16 | 0.02 |
| Model total | 0.247 | 3.319 |  | <0.001 |  |  |

* Active ROM: sum of range of motion of cervical lateral flexion (both sides), cervical rotation (both sides), neck flexion, and neck extension. Positive regression coefficients for change scores represent positive associations. NASS: North American Spine Society questionnaire; SF-36: Short Form 36 questionnaire.
