# Supplementary material for: Prognostic factors for the improvement of pain and disability following multidisciplinary rehabilitation in patients with chronic neck pain
Source: BMC Musculoskelet Disord. 2021 Apr 3;22:330. doi: 10.1186/s12891-021-04194-9 (PMC8019506; doi:10.1186/s12891-021-04194-9)
Supplement: Supplementary file 2 — Additional file 2: Table. Multivariable regression of the changes in NASS pain between baseline and programme discharge (n = 112) [file 12891_2021_4194_MOESM2_ESM.docx]

**Additional file 2:** Multivariable regression of change in NASS pain between baseline and programme discharge (*n*=112)

| Covariate | Change R^2^ | Change F-value | Regression coefficient | 95% CI | *p*-value | Bivariate correlation | Partial correlation |
| --- | --- | --- | --- | --- | --- | --- | --- |
| Constant |  |  | -1.988 | (-4.404 to 0.428) | 0.106 |  |  |
| NASS pain baseline | 0.111 | 1.547 | 0.343 | (0.167 to 0.518) | <0.001 | 0.42 | 0.36 |
| SF-36 mental health change | 0.026 | 0.087 | 0.015 | (-0.001 to 0.030) | 0.065 | 0.18 | 0.18 |
| Education | 0.023 | 0.038 | -0.136 | (-0.289 to 0.016) | 0.079 | -0.25 | -0.17 |
| SF-36 mental health baseline | 0.021 | -0.006 | 0.010 | (-0.002 to 0.022) | 0.095 | -0.04 | 0.17 |
| Gender (0=female; 1=male) | 0.012 | -0.173 | -0.265 | (-0.672 to 0.142) | 0.199 | -0.09 | -0.13 |
| Comorbidities | 0.009 | -0.237 | 0.079 | (-0.062 to 0.221) | 0.270 | 0.12 | 0.11 |
| Active ROM,* change | 0.004 | -0.329 | 0.003 | (-0.005 to 0.010) | 0.437 | 0.09 | 0.08 |
| Age | 0.003 | -0.363 | 0.006 | (-0013 to 0.024) | 0.539 | -0.05 | 0.06 |
| Active ROM,* baseline | 0.001 | -0.390 | 0.001 | (-0.004 to 0.006) | 0.655 | -0.06 | 0.05 |
| Marital Status (1=alone; 2=with partner) | 0.001 | -0.408 | 0.060 | (-0,357 to 0.477) | 0.776 | 0.07 | 0.03 |
| Model total | 0.254 | 3.444 |  |  | 0.001 |  |  |

* Active ROM: sum of range of motion of cervical lateral flexion (both sides), cervical rotation (both sides), neck flexion, and neck extension. Positive regression coefficients for change scores represent positive associations. NASS: North American Spine Society questionnaire; SF-36: Short Form 36 questionnaire.
